# Thermodynamic Modeling of the Au-Ge-X (X = In, Sb, Si, Zn) Ternary Systems

**DOI:** 10.3390/ma17092137

**Published:** 2024-05-02

**Authors:** Yuchen Bai, Qingsong Tong, Maohua Rong, Cong Tan, Xingyu Liu, Man Li, Jiang Wang

**Affiliations:** School of Materials Science and Engineering & Guangxi Key Laboratory of Information Materials, Guilin University of Electronic Technology, Guilin 541004, China; 13124132992@163.com (Y.B.); 13635644432@163.com (Q.T.); tancong0526@163.com (C.T.); liuxingyu0026@163.com (X.L.); liman560209@163.com (M.L.)

**Keywords:** Au-Ge based alloys, phase equilibria, thermodynamics, CALPHAD

## Abstract

In this study, the CALPHAD approach was employed to model the thermodynamics of the Au-Ge-X (X = In, Sb, Si, Zn) ternary systems, leveraging experimental phase equilibria data and previous assessments of related binary subsystems. The solution phases were modeled as substitutional solutions, and their excess Gibbs energies were expressed using the Redlich–Kister polynomial. Owing to the unavailability of experimental data, the solubility of the third elements in the Au-In, Au-Sb, and Au-Zn binary intermetallic compounds was excluded from consideration. Additionally, stable ternary intermetallic compounds were not reported in the literature and, thus, were not taken into account in the present thermodynamic calculations. Calculations of liquidus projections, isothermal sections, and vertical sections for these ternary systems have been performed, aligning with existing experimental findings. These thermodynamic parameters form a vital basis for creating a comprehensive thermodynamic database for Au-Ge-based alloys, which is essential for the design and development of new high-temperature Pb-free solders.

## 1. Introduction

The Pb-5wt. % Sn solder is commonly employed as a high-temperature solder in electronic packaging within the modern electronics industry. The environmental and health risks associated with lead necessitate research into high-temperature solders devoid of lead, aiming to supplant conventional solders that have high lead content. [1,2,3].

Gold-based alloys, such as Au-Sn, Au-Sb, and Au-Ge eutectic alloys, are recognized for their exceptional corrosion resistance, high electrical and mechanical strength, and superior thermal conductivity. These attributes render them highly advantageous for electronic packaging applications [4,5,6,7,8]. Au-Ge-based alloys have especially attracted much attention as high-temperature Pb-free solders [4,9]. To mitigate the costs of Au-based alloys, alloying elements, such as Ag, Bi, Cu, Ga, Ge, In, Sb and Zn, can be introduced to partially replace Au. In order to comprehensively understand the impact of alloying elements, a thorough understanding of the reliable phase equilibria and thermodynamic properties of Au-based alloys is essential. This knowledge is critical for the development of advanced high-temperature Pb-free solders based on Au, contributing to significant advancements in electronic packaging materials.

The literature [10,11,12,13,14,15,16,17,18,19,20] contains reports on thermodynamic calculations for various Au-based alloy systems, including Au-Ag-Si, Au-Ag-Pb, Au-Ge-Ni, Au-Si-Sn, Au-Bi-Sb, Au-Ge-Cu, Au-Ge-Sn, Au-Ag-Sn, Au-In-Sn, Au-Sb-Si, and Au-Ge-Sb. Despite this extensive coverage, there appears to be a lack of reported data on the thermodynamic calculations for the Au-Ge-In, Au-Ge-Si, and Au-Ge-Zn ternary systems. Although the thermodynamic model of the Au-Ge-Sb system by Wang et al. [15] aligns well with experimental outcomes, recent updates have been made to the thermodynamic parameters of its binary subsystems [21]. Consequently, there is an ongoing need to re-optimize the Au-Ge-Sb ternary system. Therefore, the objective of this study was to conduct thermodynamic calculations of phase equilibria in the Au-Ge-X (X = In, Sb, Si, Zn) ternary systems using the CALPHAD method. This research serves as a fundamental step toward establishing a comprehensive thermodynamic database for multicomponent Au-based alloy systems.

## 2. Literature Information

### 2.1. The Au-Ge-In Ternary System

The literature by Okamoto et al. [22] and Chevalier [23] provides comprehensive reviews of the Au-Ge system. Subsequently, it was re-optimized by Wang et al. [14] using the lattice stability parameters updated by Dinsdale [24]. The optimization outcomes of the Au-Ge system [14] are in accordance with the experimental findings, encompassing phase diagrams and thermodynamic properties. A thermodynamic assessment of the Au-In system was performed by Ansara and Nabot [25]. Liu et al. [26] re-optimized it, taking into account the temperature dependence of the measured enthalpy of mixing of the liquid phase. The Ge-In system was assessed thermodynamically by Chewvalier et al. [27]. The calculated results of the Au-In and Ge-In systems [26,27] demonstrate good agreement with the experimental findings. The thermodynamic parameters of the Au-Ge, Au-In, and Ge-In systems obtained by Wang et al. [14], Liu et al. [26], and Chevalier et al. [27] were directly employed in the current calculation of the Au-Ge-In ternary system. 

The Au-Ge-In system was studied experimentally by Butt [28], while the thermodynamic properties of this ternary system were not reported in the literature. The experimental results [28] show that two E-type invariant reactions (liquid ↔ diamond(Ge) + AuIn + AuIn_2_ at 744 K, liquid ↔ diamond(Ge) + AuIn_2_ + tetragonal(In) at 429 K) were determined, and four vertical sections of AuIn-Ge, Au_41.5_In_58.5_-Ge, AuIn_2_-Ge, and Au_20_In_80_-Ge were measured. The stable ternary intermetallic compounds in this ternary system were not observed by Butt [28]. The experimental results [28] were taken into account in the present calculation.

### 2.2. The Au-Ge-Sb Ternary System

The Au-Sb system, a pivotal component of the Au-Ge-Sb system, has been extensively investigated by numerous researchers [19,29,30,31,32]. Liu et al. [31] conducted an optimization of the Au-Sb system, taking into account the existing experimental data. The phase diagram and thermodynamic data previously published align well with the recent calculations [30]. The optimization of the Ge-Sb system was initially conducted by Wang et al. [15] and Chewvalier et al. [27]. Due to the oversight regarding the solubility of Ge in the rhombohedral Sb phase in these earlier studies, Liu et al. [21] conducted a re-optimization of the Ge-Sb system. The re-optimized results [21] correlate closely with the experimental observations. Consequently, the refined thermodynamic data for the Ge-Sb system from Liu et al. [21], along with the Au-Sb system data from Liu et al. [31], have been incorporated into the current calculations for the Au-Ge-Sb system. 

Zwingmann [33] conducted a study on the phase equilibria of the Au-Ge-Sb system; however, the thermodynamic properties of this system have not been reported in the literature. Prince et al. [34] conducted a review of the Au-Ge-Sb system as part of their compilation of Au-based alloy phase diagrams. According to the experimental results [33], the Au-Ge-Sb system contains two invariant reactions: the peritectic reaction (U), liquid + rhombohedral (Sb) ↔ diamond (Ge) + AuSb_2_ at 703 K and the eutectic reaction (E), liquid ↔ diamond (Ge) + fcc (Au) + AuSb_2_ at 561 K. Three vertical sections of AuSb_2_-Ge, Ge_0.1_Sb_0.9_-Au, and 15 at.% Ge were measured by Zwingmann [33]. Zwingmann [33] did not observe the presence of stable ternary intermetallic compounds in the Au-Ge-Sb system.

The Au-Ge-Sb system was optimized by Wang et al. [15]. Afterward, Liu et al. [21] updated the thermodynamic parameters of the Ge-Sb system. Liu et al. [21] took into account the solubility of Ge in rhombohedral(Sb) during the optimization of the Ge-Sb system. The thermodynamic assessment of the Au-Ge-Sb system by Wang et al. [15] was found to be in good agreement with the experimental data [33]. However, updated thermodynamic parameters for the Ge-Sb systems were provided by Liu et al. [21], indicating a necessity to re-optimize the Au-Ge-Sb system based on the findings reported in reference [33].

### 2.3. The Au-Ge-Si Ternary System

Meng et al. [20] conducted optimization studies on the Au-Si system, while Olesinski and Abbaschian [35] and Bergman et al. [36] focused on reviewing and optimizing the Ge-Si system, respectively. The optimization results for the Au-Si and Ge-Si systems from references [20,36] were well-aligned with the experimental findings and were, therefore, integrated into the current modeling of the Au-Ge-Si system.

While the literature does not detail the thermodynamic properties of the Au-Ge-Si system, Predel et al. [37] have explored its phase equilibria using thermal and metallographic analyses. They identified four vertical sections, namely Au-Ge_0.20_Si_0.80_, Au-Ge_0.40_Si_0.60_, Au-Ge_0.60_Si_0.40_, and Au-Ge_0.80_Si_0.75_, and constructed the liquidus projection for this system. The experimental findings from Predel et al. [37] have been incorporated into the ongoing calculations for the Au-Ge-Si system.

### 2.4. The Au-Ge-Zn Ternary System

The Au-Zn system was assessed by Okamoto and Massalski and Krachler et al. [38,39]. Liu et al. [40] provided an updated thermodynamic assessment of the Au-Zn system. The thermodynamic calculation of the Ge-Zn system was performed by Chewvalier et al. [27]. The calculated results of the Au-Zn and Ge-Zn systems by Liu et al. [40] and Chewvalier et al. [27] are in accordance with experimental data and were consequently utilized in the present calculation of the Au-Ge-Zn system. 

As for the phase equilibria of the Au-Ge-Zn system, the vertical section of AuZn-Ge was investigated by Butt et al. [41] using differential thermal analysis (DTA) and the metallographic technique. Their experimental vertical section shows a pseudo-binary eutectic characteristic, and the eutectic composition and temperature were determined to be 12.2 ± 0.2 at.% Ge and 946 K, respectively. At the eutectic temperature, the solubility of Ge in the AuZn alloy was determined to be 1.3 atomic percent Ge, as reported in reference [41]. These experimental findings [41] were utilized in the current calculations.

## 3. Thermodynamic Models

### 3.1. Solution Phases

The solution phase φ is described by substituting the solution model. It includes liquid, diamond(Ge, Si), tetragonal(In), hcp(Zn), fcc(Au), α(Au, In), hcp(Au, In), rhombohedral(Sb), and ε_1_-Au_3_Zn_17_ in the Au-Ge-X (X = In, Sb, Si, Zn) systems. The molar Gibbs energy of the solution phase φ is denoted as:

(1) Gmφ=∑i=Au,Ge,XxiGiφ0+RT∑i=Au,Ge,Xxilnxi+GmφE

The mole fraction of each element i(Au, Ge, In, Sb, Si, Zn) is denoted as x_i_. The molar Gibbs energy for the phase ϕ of each element, extracted from the SGTE database [24], is represented as Giφ0. The constant R represents the gas constant, and T symbolizes the absolute temperature measured in Kelvin. The excess Gibbs energy for phase GmφE is articulated through the Redlich–Kister–Muggianu polynomial, as documented in reference [42,43]:

(2) GmφE=xAuxGe∑j=0nLAu,Geφ(j)xAu−xGej+xAuxX∑j=0nLAu,Xφ(j)xAu−xXj+xGexX∑j=0nLGe,Xφ(j)xGe−xXj+xAuxGexXLAu,Ge,Xφ

(3) LAu,Ge,Xφ=xAuLAu,Ge,X(0)+xGeLAu,Ge,X(1)+xXLAu,Ge,X(2)

(4) LAu,Ge,X(j)=Ai+BiT

The binary interaction parameters, LAu,Geφ(j), LAu,Xφ(j), and LGe,Xφ(j) (X = In, Sb, Si, Zn) were taken from the Au-Ge, Au-In, Au-Sb, Au-Si, Au-Zn, Ge-In, Ge-Sb, Ge-Si, and Ge-Zn systems assessed by Wang et al. [14], Liu et al. [26], Liu et al. [31], Meng et al. [20], Liu et al. [40], Chewvalier et al. [27], Liu et al. [21], and Bergman et al. [36], respectively. The ternary interaction parameters, LAu,Ge,In(j), LAu,Ge,Sb(j), LAu,Ge,Si(j), and LAu,Ge,Zn(j) (j = 0, 1, 2) are to be assessed in this work.

### 3.2. Intermetallic Compounds

Nine Au-In intermetallic compounds (α(Au, In), β-Au_4_In, β′-Au_4_In, γ-Au_9_In_4_, γ′-Au_7_In_3_, ψ-Au_3_In_2_, Au_3_In, AuIn, and AuIn_2_), one Au-Sb intermetallic compound (AuSb_2_), and eleven Au-Zn intermetallic compounds (α_1_-Au_3_Zn, α_2_-Au_3_Zn, α_3_-Au_3_Zn, ε_1_-Au_3_Zn_17_, ε_2_-Au_3_Zn_17_, β_1_-AuZn, Au_5_Zn_3_, δ_1_-Au_11_Zn_14_, γ_1_-AuZn_3_, γ_2_-AuIn_3_ and γ_3_-AuZn_3_) are stable in the Au-In, Au-Sb, and Au-Zn systems. There are no stable ternary intermetallic compounds in the Au-Ge-X (X = In, Sb, Si, Zn) systems. The crystal structure data of the solid solution phases in the Au-Ge-X (X = In, Sb, Si, Zn) systems are shown in Table 1.

Based on the calculated results of the Au-In, Au-Sb, and Au-Zn systems by Liu et al. [26], Wang et al. [31], and Liu et al. [40], the molar Gibbs energies of these intermetallic compounds (β-Au_4_In, β′-Au_4_In, γ′-Au_7_In_3_, Au_3_In, AuIn, AuIn_2_, AuSb_2_, α_2_-Au_3_Zn, ε_2_-Au_3_Zn_17_, β_1_-AuZn, Au_5_Zn_3_, δ-Au_11_Zn_14_, and γ_2_-AuIn_3_) are described by the two-sublattice model, while those of γ-Au_9_In_4_, ψ-Au_3_In_2_, α_1_-Au_3_Zn, α_3_-Au_3_Zn, and γ_3_-AuZn_3_ are expressed by the three-sublattice model. In this research, the molar Gibbs energy of the γ_1_-AuZn_3_ phase is modeled using a four-sublattice approach, whereas the α(Au, In) and ε_1_-Au_3_Zn_17_ intermetallic compounds are characterized through the substitutional solution model. Owing to an absence of experimental data, the solubility of Ge within the Au-In, Au-Sb, and Au-Zn intermetallic compounds has not been included in the thermodynamic assessments for the Au-Ge-In, Au-Ge-Sb, and Au-Ge-Zn systems. The Gibbs energy values for these binary intermetallic compounds are derived from studies by Liu et al. [26,31,40]. Thermodynamic models of these Au-In, Au-Sb, and Au-Zn binary intermetallic compounds were shown completely in Refs. [26,31,40] and, thus, are not duplicated here.

## 4. Calculated Results and Discussion

Leveraging the lattice stabilities compiled by Dinsdale [24], the optimization of thermodynamic parameters for the Au-Ge-X (X = In, Sb, Si, Zn) systems was performed using the PARROT module within the Thermo-Calc^®^ software 2024a, as developed by Sundman et al. [44]. The final results, detailing the thermodynamic parameters for all phases within these systems, are comprehensively presented across Table 2, Table 3, Table 4 and Table 5.

### 4.1. The Au-Ge-In System

**Figure 1:**
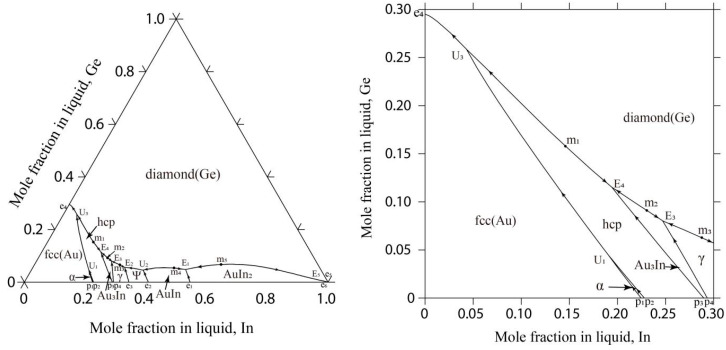
The calculated liquidus projection of the Au-Ge-In system in this study.

**Figure 2:**
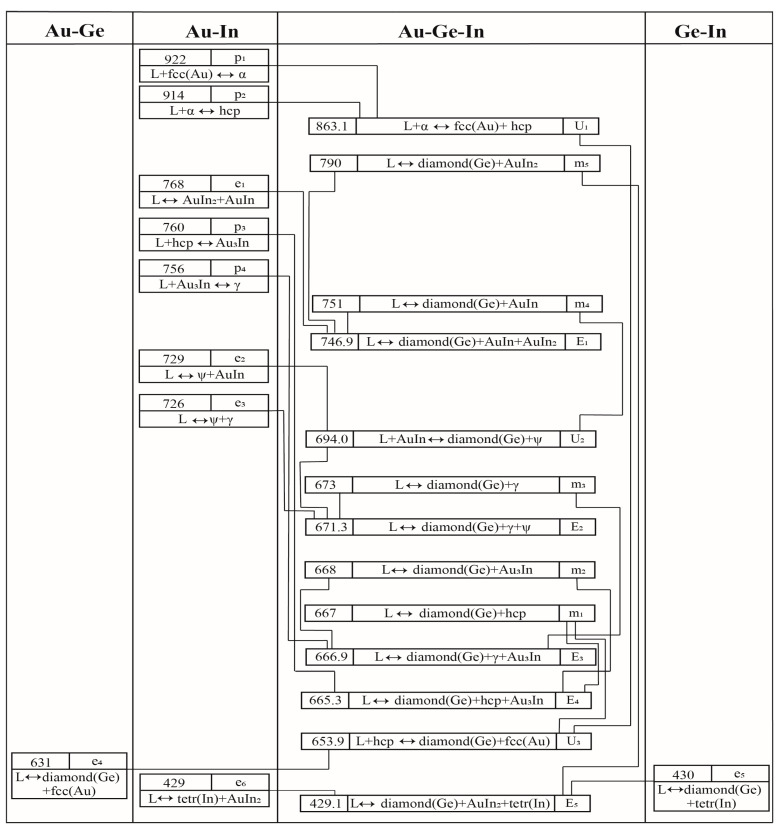
Reaction scheme of the Au-Ge-In system.

In this study, Figure 1 depicts the liquidus projection for the Au-Ge-In system. Additionally, Figure 2 outlines the reaction scheme for this system. 

It can be seen in Figure 1 that there are five E-type invariant reactions (E_1_: Liquid ↔ diamond(Ge) + AuIn + AuIn_2_ at 747 K, E_2_: Liquid ↔ diamond(Ge) + γ-Au_9_In_4_ + ψ-Au_3_In_2_ at 671 K, E_3_: Liquid ↔ diamond(Ge) + γ-Au_9_In_4_ + Au_3_In at 667 K, E_4_: Liquid ↔ diamond(Ge) + hcp(Au, In) + Au_3_In at 665 K, E_5_: Liquid ↔ diamond(Ge) + AuIn_2_ + tetragonal(In) at 429 K) and three U-type invariant reactions (U_1_: Liquid + α(Au, In) ↔ fcc(Au) + hcp(Au, In) at 863 K, U_2_: Liquid + AuIn ↔ diamond(Ge) + ψ-Au_3_In_2_ at 694 K, U_3_: Liquid + hcp(Au, In) ↔ diamond(Ge) + fcc(Au) at 654 K). The calculated temperature and composition of E_1_ (747 K, 44.4 at.% Au-4.8 at.% Ge) are in good accordance with the experimental results (744 K, 43.3 at.% Au-3.5 at.% Ge) [28]. The calculated temperature of E_5_ in the rich In part is 429 K, which is well consistent with the reported value (429 K) [28]. Furthermore, it was observed in Figure 1 and Figure 2 that there are five maximum points (m_1_: 667 K, 69.6 at.% Au-15.8 at.% Ge; m_2_: 668 K, 67.9 at.% Au-9.1 at.% Ge; m_3_: 673 K, 64.9 at.% Au-6.3 at.% Ge; m_4_: 751 K, 47.4 at.% Au-5.3 at.% Ge; m_5_: 790 K, 31.0 at.% Au-6.7 at.% Ge) located on the monovariant curves of U_3_-E_4_, E_4_-E_3_, E_3_-E_2_, U_2_-E_1_, and E_1_-E_5_, which needs to be verified by the further experimental investigations.

Table 6 displays the results from the invariant reactions in the Au-Ge-In system, including temperatures and compositions, which align with the experimental findings detailed in reference [28].

**Figure 3:**
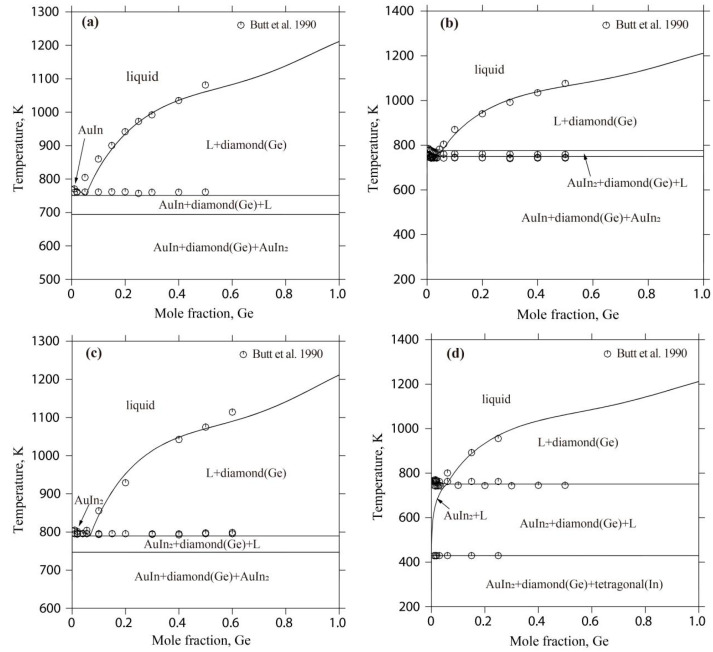
Calculated vertical sections of the Au-Ge-In system with the experimental results [28]: (**a**) AuIn-Ge; (**b**) Au_41.5_In_58.5_-Ge; (**c**) AuIn_2_-Ge; and (**d**) Au_20_In_80_-Ge.

Figure 3 compares the calculated four vertical sections of AuIn-Ge, Au_41.5_In_58.5_-Ge, AuIn_2_-Ge, and Au_20_In_80_-Ge with the experimental results [28]. As can be seen, these four vertical sections display a pseudo-binary eutectic characteristic. In Figure 3a, the calculated transition temperature and the composition of the liquid phase for the AuIn-Ge vertical section are 764 K and 3.0 at.% Ge, which are consistent with the experimental results (761 K and 2.0 at.% Ge) [28], respectively. 

Specifically, in panel (a) of Figure 3, the transition temperature and the composition of the liquid phase for the AuIn-Ge section were determined to be 764 K and 3.0 atomic percent Ge, aligning closely with the experimental values of 761 K and 2.0 atomic percent Ge [28]. Panels (b), (c), and (d) of Figure 3 show that the results for the Au_41.5_In_58.5_-Ge, AuIn_2_-Ge, and Au_20_In_80_-Ge sections also correspond well with the experimental findings [28]. The calculated liquidus in these sections within the Au-Ge-In system demonstrates good agreement with the documented experimental results [28].

### 4.2. The Au-Ge-Sb System

**Figure 4:**
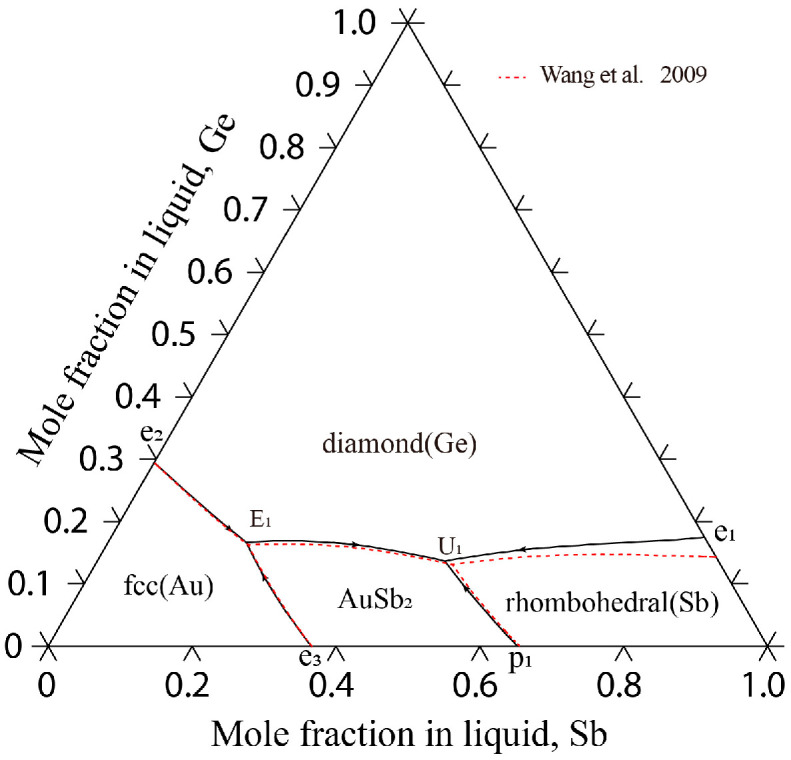
Calculated liquidus projection of the Au-Ge-Sb system in this study with the calculated results by Wang et al. [15].

**Figure 5:**
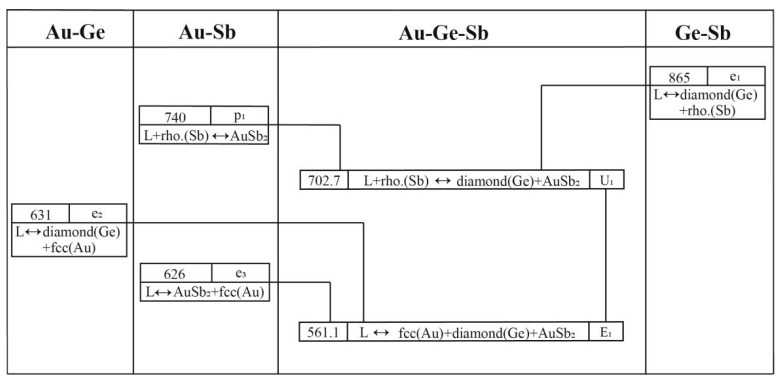
Reaction scheme of the Au-Ge-Sb system.

Figure 4 displays the liquidus projection for the Au-Ge-Sb system calculated in this study, alongside comparisons to the projections made by Wang et al. [15]. Figure 5 depicts the reaction scheme derived from the current calculations for the same system. Additionally, Table 7 lists the temperatures and compositions for the invariant reactions within the Au-Ge-Sb system, validated against both experimental findings [33] and previous calculations [15]. As shown in Figure 4, the liquidus projection of the Au-Ge-Sb system includes one E-type invariant reaction (E_1_: Liquid ↔ diamond(Ge) + fcc(Au) + AuSb_2_) and one U-type invariant reaction (U_1_: Liquid + rhombohedra(Sb) ↔ diamond(Ge) + AuSb_2_). In this work, the temperatures of these two invariant reactions were calculated to be 561 K and 703 K, which are consistent well with the experimental data (561 K and 703 K) measured by Zwingmann [33]. The calculated results of this work are much better than the results (560 K and 702 K) of Wang et al. [15]. Moreover, the compositions of the liquid phase for these two invariant reactions were calculated to be 64.1 at.% Au-16.6 at.% Ge and 38.0 at.% Au-13.7 at.% Ge, which are consistent with the experimental data (68.0 at.% Au-15.0 at.% Ge and 35.0 at.% Au-14.0 at.% Ge) [33] and the calculated results [15].

**Figure 6:**
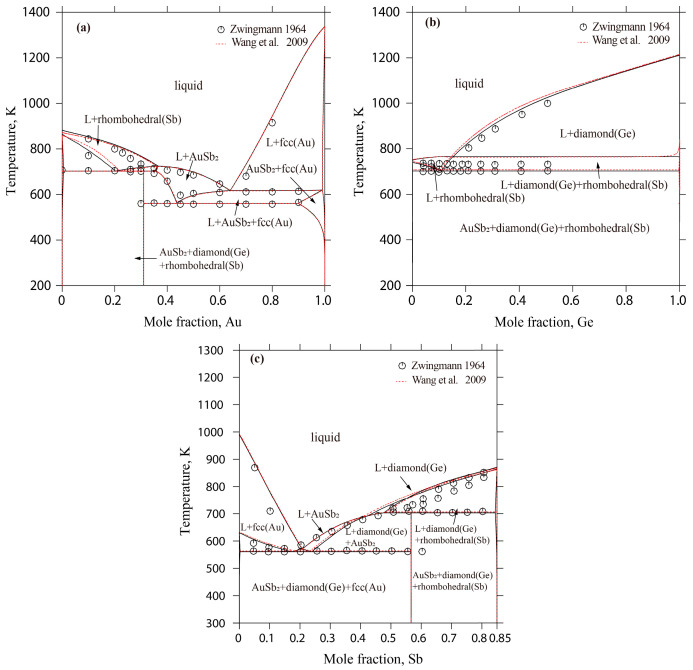
Calculated vertical sections of the Au-Ge-Sb system with the experimental results [33] and the calculated results [15]: (**a**) Au-Ge_0.1_Sb_0.9_; (**b**) AuSb_2_-Ge; and (**c**) 15 at.% Ge.

Figure 6 presents the calculated vertical sections of Au-Ge_0.1_Sb_0.9_, AuSb_2_-Ge, and 15 at.% Ge, compared against both the experimental findings [33] and the previously calculated results [15]. Panels (a) and (c) of Figure 6 reveal that the phase-transition temperatures and liquidus lines for the Au-Ge_0.1_Sb_0.9_ and 15 atomic percent Ge sections align closely with the experimental data [33]. For the AuSb_2_-Ge section, the calculated liquidus corresponds with the calculated data [15] but exhibits a minor variance from the experimental observations [33].

### 4.3. The Au-Ge-Si Ternary System

**Figure 7:**
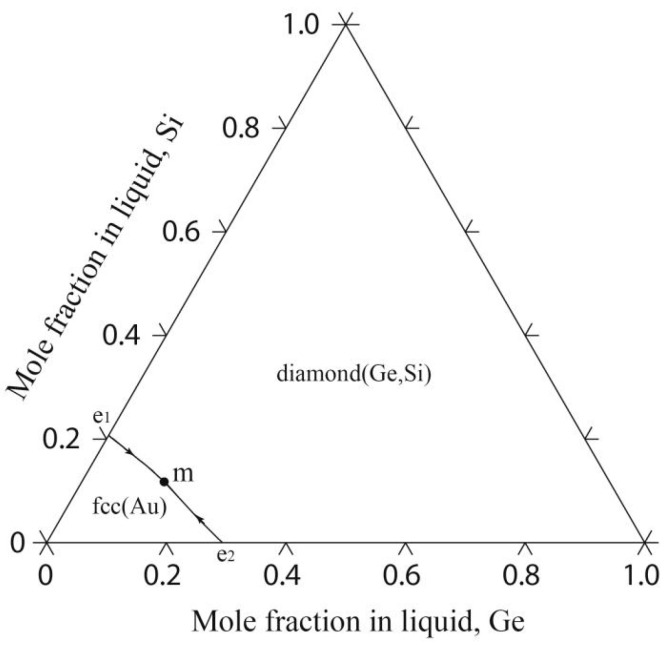
Calculated liquidus projection of the Au-Ge-Si system in this study.

The calculated liquidus projection of the Au-Ge-Si system is shown in Figure 7; the Au-Ge-Si system presents a monotropic eutectic reaction including the liquid phase, fcc(Au) and diamond(Ge, Si). The monovariant curve from the Au-Si binary eutectic point e_1_ to the Au-Ge binary eutectic point e_2_ passes smoothly through a minimum point (599 K and 13.8 at.% Ge-11.7 at.% Si), which is consistent with the reported data (599 K and 8.8 at.% Ge-13.2 at.% Si) by Predel et al. [37].

**Figure 8:**
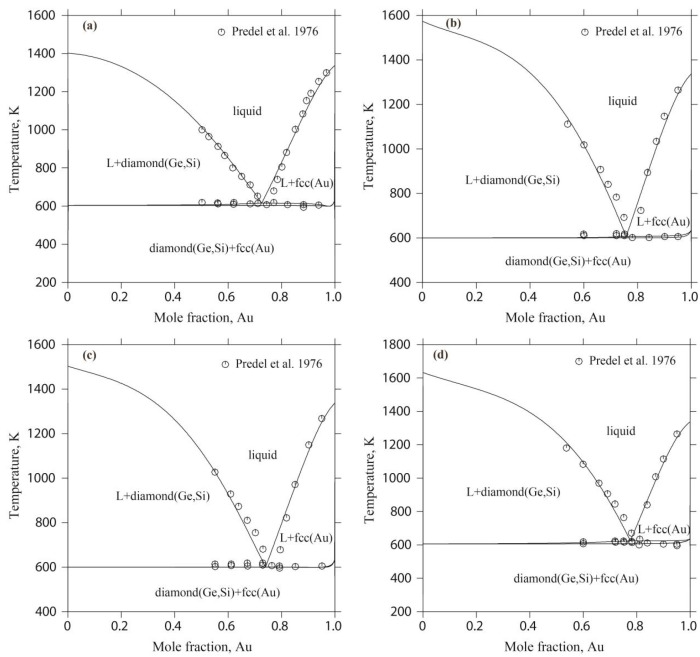
Calculated vertical sections of the Au-Ge-Si system with the experimental results [37]: (**a**) Au-Ge_0.8_Si_0.2_; (**b**) Au-Ge_0.4_Si_0.6_; (**c**) Au-Ge_0.6_Si_0.4_; and (**d**) Au-Ge_0.2_Si_0.8_.

Comparing the experimental data and the calculated results of the four vertical sections of Au-Ge_0.8_Si_0.2_, Au-Ge_0.6_Si_0.4_, Au-Ge_0.4_Si_0.6_, and Au-Ge_0.2_Si_0.8_, the calculated liquidus temperatures and phase-transition temperatures for the four vertical sections match the experimental data well, as shown in Figure 8. All the four vertical sections present a pseudo-binary eutectic characteristic [37].

### 4.4. The Au-Ge-Zn Ternary System

**Figure 9:**
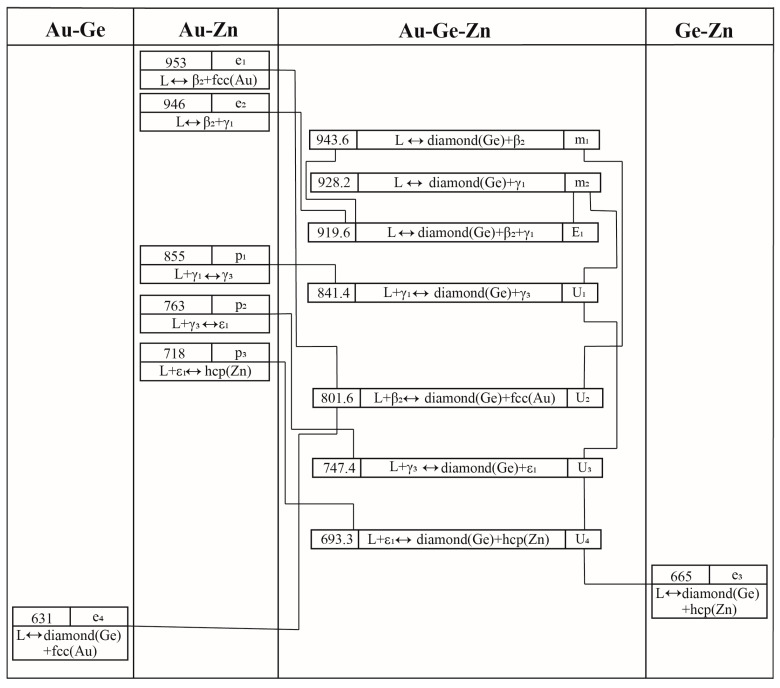
Reaction scheme of the Au-Ge-Zn system.

**Figure 10:**
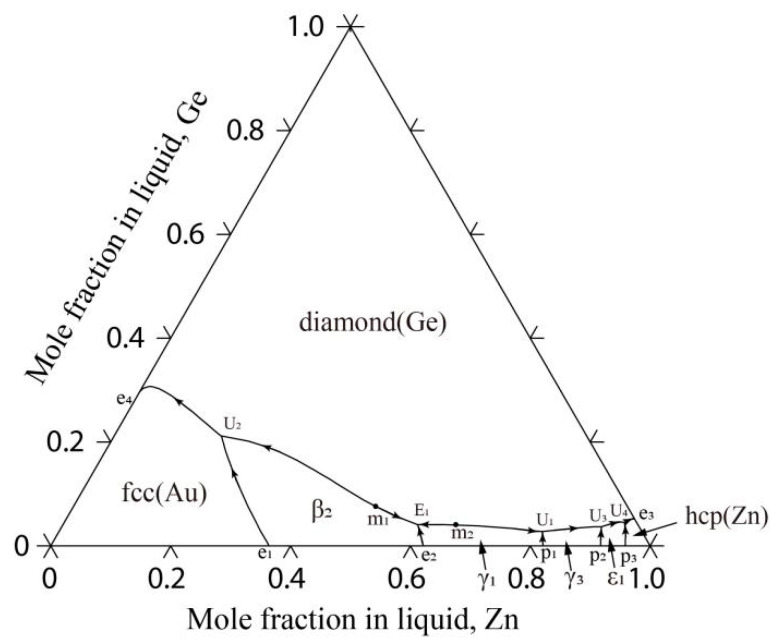
Calculated liquidus projection of the Au-Ge-Zn system in this study.

Figure 9 illustrates the reaction scheme of this system as obtained in this study, and Figure 10 shows the liquidus projection of the Au-Ge-Zn system that was calculated. Table 8 provides a summary of the temperatures and compositions of the invariant reactions.

It is obvious that the liquidus projection of the Au-Ge-Zn system contains one E-type invariant reaction (E_1_: Liquid ↔ β_2_-AuZn + diamond(Ge) + γ_1_-AuZn_3_ at 920 K) and four U-type invariant reactions (U_1_: Liquid + γ_1_-AuZn_3_ ↔ diamond(Ge) + γ_3_-AuZn_3_ at 841 K, U_2_: Liquid + β_2_-AuZn ↔ diamond(Ge) + fcc (Au) at 802 K, U_3_: Liquid + γ_3_-AuZn_3_ ↔ diamond(Ge) + ε_1_-Au_3_Zn_17_ at 747 K, U_4_: Liquid + ε_1_-Au_3_Zn_17_ ↔ diamond(Ge) + hcp (Zn) at 693 K). Moreover, the liquidus projection of the Au-Ge-Zn system illustrates that the presence of two maximum points (m_1_: 944 K and 44.0 at.% Au-9.4 at.% Ge; m_2_: 928 K and 31.9 at.% Au-4.1 at.% Ge) that are located on the monovariant curves of E_1_-U_2_ and E_1_-U_1_. Because of the absence of experimental information on the liquidus projection of the Au-Ge-Zn system, additional experiments need to be performed to determine these invariant reactions and maximum points in the monovariant curves, which would be used to confirm the present calculations.

**Figure 11:**
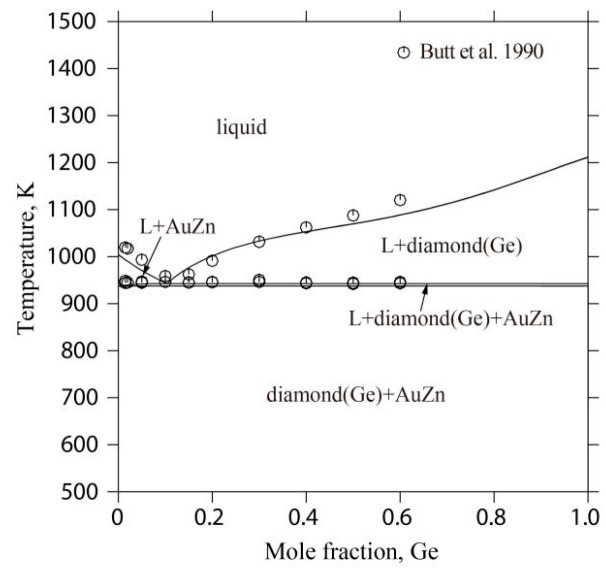
Calculated vertical sections of the Au-Ge-Zn system with the experimental results [41].

Figure 11 illustrates the computed vertical section of the AuZn-Ge system alongside the documented experimental outcomes [41]. This section exhibits characteristics of a pseudo-binary eutectic system. The study determined the eutectic reaction’s temperature and composition at 943 K and 10.4 atomic percent Ge, aligning closely with the experimental measurements of 946 K and 12.2 atomic percent Ge [41]. Notably, the alignment of the calculated liquidus for this section with the experimental data [41] demonstrates the model’s accuracy.

## 5. Conclusions

This study employed the CALPHAD approach, integrating it with data from the literature reviews and previous binary subsystem evaluations to conduct thermodynamic modeling of the Au-Ge-X (X = In, Sb, Si, Zn) systems. The comparison of the calculated liquidus projections and vertical sections of the Au-Ge-X (X = In, Sb, Si, Zn) systems demonstrates that the present calculations are consistent with the reported experimental data. Finally, a set of available and self-consistent thermodynamic parameters of the Au-Ge-X (X = In, Sb, Si, Zn) systems was obtained. These parameters enable the prediction of phase equilibria and thermodynamic behaviors of the Au-Ge-X alloys. Such data are crucial for the development of a thermodynamic database for multi-component Au-based alloys, enhancing the exploration of innovative high-temperature lead-free solders for electronic applications.

## Figures and Tables

**Table 1:** Crystallographic data of the solid solution phases in the Au-Ge-X (X = In, Sb, Si, Zn) systems.

Phase	Prototype	Space Group	Pearson Symbol	Thermodynamic Model	Reference
fcc (Au)	Cu	Fm$\bar{3}$m	cF4	(Au)	[26]
diamond (Ge/Si)	Diamond	Fd$\bar{3}$m	cF8	(Ge)/(Si)	[35]
tetragonal (In)	In	I4/mmm	tI2	(In)	[26]
rhombohedral (Sb)	As	R$\bar{3}$m	hR6	(Sb)	[31]
hcp (Zn)	Mg	P6_3_/mmc	hP2	(Zn)	[40]
hcp (Au, In)	Mg	P6_3_/mmc	hP2	(Au, In)	[26]
α (Au, In)	Ni_3_Ti	P6_3_/mmc	hP16	(Au, In)	[26]
β-Au_4_In	Cu_11_Sb_3_	Amm2	—	(Au)_0.785_: (In)_0.215_	[26]
β′-Au_4_In	Cu_10_Sb_3_	—	—	(Au)_0.77778_: (In)_0.22222_	[26]
γ-Au_9_In_4_	Cu_9_Al_4_	P$\bar{4}$3m	cP52	(Au)_0.69231_: (Au, In)_0.23077_: (In)_0.07692_	[26]
γ′-Au_7_In_3_	Au_7_In_3_	P3	hP60	(Au)_0.7_: (In)_0.3_	[26]
ψ-Au_3_In_2_	Ni_2_Al_3_	P3m1	hP5	(Au)_0.5_: (Au, In)_0.33333_: (In)_0.16667_	[26]
Au_3_In	Cu_3_Ti	Pmmn	oP8	(Au)_0.75_: (In)_0.25_	[26]
AuIn	—	—	—	(Au)_0.5_: (In)_0.5_	[26]
AuIn_2_	CaF_2_	Fm$\bar{3}$m	cF12	(Au)_0.33333_: (In)_0.66667_	[26]
AuSb_2_	FeS_2_	Pa3	cP12	(Au)_0.3333_: (Sb)_0.6667_	[31]
α_1_-Au_3_Zn	Ag_3_Mg	—	—	(Au)_0.6_: (Au, Zn)_0.2_: (Zn)_0.2_	[40]
α_2_-Au_3_Zn	—	Abam (Cmca)	oC32	(Au)_0.75_: (Zn)_0.25_	[40]
α_3_-Au_3_Zn	Cu_3_Pd	Pn2n/Pnmn	—	(Au)_0.64286_: (Au, Zn)_0.25_: (Zn)_0.10714_	[40]
ε_1_-Au_3_Zn_17_	Mg	P6_3_/mmc	hP2	(Au, Zn)	[40]
ε_2_-Au_3_Zn_17_	—	—	—	(Au)_0.15_: (In)_0.85_	[40]
β_1_-AuZn	CsCl	Pm$\bar{3}$m	cP2	(Au, Zn)_0.5_: (Au, Zn)_0.5_	[40]
Au_5_Zn_3_	—	Ibam	—	(Au)_0.625_: (Zn)_0.375_	[40]
δ_1_-Au_11_Zn_14_	—	—	—	(Au)_0.44_: (Zn)_0.56_	[40]
γ_1_-AuZn_3_	Cu_5_Zn_8_	—	—	(Au, Zn)_0.15385_: (Au)_0.15385_: (Au, Zn)_0.23077_: (Zn)_0.46153_	[40]
γ_2_-AuZn_3_	H_3_U	Pn$\bar{3}$m	cP32	(Au)_0.25_: (Zn)_0.75_	[40]
γ_3_-AuZn_3_	—	—	—	(Au)_0.12_: (Au, Zn)_0.16_: (Zn)_0.72_	[40]

**Table 2:** Thermodynamic parameters for the Au-Ge-In system.

Phase	Thermodynamic Parameters	Reference
liquid (Au, Ge, In)	LAu,Geliq 0=−18,294.684−13.671T LAu,Geliq= 1−8894.639−6.339T	[14]
	LAu,Geliq 2=−2174.476−4.925T	[14]
	LAu,Inliq 0=−76,196.19+64.291T−6.638TlnT	[26]
	LAu,Inliq 1=−31,134.02+81.358T−8.513TlnT	[26]
	LGe,Inliq= 0+1587.2−0.3871T LGe,Inliq= 1−583.5−1.511T	[26]
	LAu,Ge,Inliq 0=+62,816.812 LAu,Ge,Inliq 1=+53,443.685 LAu,Ge,Inliq= 2−23,278.378	This work
fcc (Au, Ge, In)	LAu,Gefcc=+10,198.859−23.114T 0	[14]
	LAu,Infcc= 0−48,493.65+46.624T−6.831TlnT	[26]
	LAu,Infcc=+498.45 1	[26]
diamond(Ge)	GGedia. 0 cited from SGTE database	[14]
tetragonal(In)	GIntetr. 0 cited from SGTE database	[26]
hcp(Au, In)	LAu,Inhcp= 0−55,780.55+13.820T LAu,Inhcp= 1+6788.95−32.894T	[26]
α(Au, In)	LAu,Inα= 0−48,238.66+5.355T LAu,Inα=−48.36−16.793T 1	[26]
β-Au_4_In	GAu:Inβ-Au4In=−8980.42−3.304T+0.785G0Aufcc+0.215GIntetr.0	[26]
β′-Au_4_In	GAu:Inβ′-Au4In=−9382.52−3.102T+0.778G 0Aufcc+0.222GIntetr. 0	[26]
γ-Au_9_In_4_	GAu:Au:Inγ-Au9In4=−2830.47−2.519T+0.923G 0Aufcc+0.077GIntetr. 0	[26]
	GAu:In:Inγ-Au9In4=−11,992.16−3.651T+0.692G 0Aufcc+0.308GIntetr. 0	[26]
	$L_{Au:Au,In:In}^{\gamma -Au_{9}In_{4}}=+2144.6$	[26]
γ′-Au_7_In_3_	GAu:Inγ′-Au7In3=−12,813.11−2.054T+0.7G 0Aufcc+0.3GIntetr. 0	[26]
ψ-Au_3_In_2_	GAu:Au:Inψ-Au3In2=+2153.38−8.039T+0.833G 0Aufcc+0.167GIntetr. 0	[26]
	GAu:In:Inψ-Au3In2=−18,225.14−3.0T+0.5G 0Aufcc+0.5GIntetr. 0	[26]
	$L_{Au:Au,In:In}^{\psi -Au_{3}In_{2}}=-15,683.16$	[26]
Au_3_In	GAu:InAu3In=−10,582.67−2.932T+0.75G 0Aufcc+0.25GIntetr. 0	[26]
AuIn	GAu:InAuIn=−20,188.37+2.379T+0.5G 0Aufcc+0.5GIntetr. 0	[26]
AuIn_2_	GAu:InAuIn2=−26,129.06+11.113T+0.333G 0Aufcc+0.667GIntetr. 0	[26]

**Table 3:** Thermodynamic parameters for the Au-Ge-Sb system.

Phase	Thermodynamic Parameters	Reference
liquid (Au, Ge, Sb)	LAu,Geliq 0=−18,294.684−13.671T LAu,Geliq= 1−8894.639−6.339T	[14]
	LAu,Geliq 2=−2174.476−4.925T	[14]
	LAu,Sbliq 0=−15,067.47+23.15424T−4.988235TlnT	[31]
	LAu,Sbliq 1=−2427.37−8.3278T	[31]
	LGe,Sbliq= 0+3289.7−0.521T	[21]
	LAu,Ge,Sbliq 0=−22,395.305	This work
	LAu,Ge,Sbliq 1=+11,801.969	This work
	LAu,Ge,Sbliq= 2+17,008.798	This work
fcc (Au, Ge, Sb)	LAu,Gefcc=+10,198.859−23.114T 0	[14]
	LAu,Sbfcc= 0+24,512.703−25T	[31]
diamond (Ge, Sb)	LGe,Sbdia.= 0+79,210.1−19.8T	[21]
rhombohedral (Ge, Sb)	LAu,Sbrho.= 0+3000−0.1T	[31]
	LGe,Sbrho.= 0+10,695−6.557T	[21]
AuSb_2_	GAu:SbAuSb2=−5450.31+12.8064T−1.63691TlnT+0.333G 0Aufcc+0.667GSbrho. 0	[31]

**Table 4:** Thermodynamic parameters for the Au-Ge-Si system.

Phase	Thermodynamic Parameters	Reference
liquid (Au, Ge, Si)	LAu,Geliq 0=−18,294.684−13.671T	[14]
	LAu,Geliq= 1−8894.639−6.339T	[14]
	LAu,Geliq 2=−2174.476−4.925T	[14]
	LAu,Siliq 0=−24,103.303−15.139T	[20]
	LAu,Siliq 1=−29,375.278+1.107T	[20]
	LAu,Siliq= 2−13,032.241	[20]
	LGe,Siliq= 0+6000	[35]
	LAu,Ge,Siliq 0=−26,000	This work
	LAu,Ge,Siliq 1=+70,000	This work
	LAu,Ge,Siliq= 2+48,000	This work
fcc (Au, Ge, Si)	LAu,Gefcc=+10,198.859−23.114T 0	[14]
	LAu,Sifcc=+2000 0	[20]
diamond (Au, Ge, Si)	LAu,Sidia.= 0+40,000	[20]
	LGe,Sidia.= 0+3500	[35]

**Table 5:** Thermodynamic parameters for the Au-Ge-Zn system.

Phase	Thermodynamic Parameters	Reference
liquid (Au, Ge, Zn)	LAu,Geliq 0=−18,294.684−13.671T, LAu,Geliq= 1−8894.639−6.339T,LAu,Geliq 2=−2174.476−4.925T	[14]
	LAu,Znliq 0=−96,492.26+42.713T−3.041TlnT, LAu,Znliq 1=−5576.71+0.015T	[40]
	LGe,Znliq= 0+4940.9−6.510T, LGe,Znliq= 1+1119	[27]
	LAu,Ge,Znliq 0=+10,000, LAu,Ge,Znliq 1=+50,000, LAu,Ge,Znliq= 2+100,000	This work
fcc (Au, Ge, Zn)	LAu,Gefcc=+10,198.859−23.114T 0	[14]
	LAu,Znfcc= 0−95,112.59+101.687T−11.897TlnT, LAu,Znfcc=+452.29+7.540T 1	[40]
Diamond (Ge, Zn)	LGe,Zndia.= 0+80T	[27]
hcp(Au, Zn)	LAu,Znhcp= 0−49,193.15+11.770T, LAu,Znhcp= 1+21,680.4	[40]
α_1_-Au_3_Zn	GAu:Au:Znα1-Au3Zn=−78,040.1+11.236T+4G 0Aufcc+GZnhcp 0	[40]
	GAu:Zn:Znα1-Au3Zn=−120,613.65+31.264T+3G 0Aufcc+2GZnhcp 0	[40]
	$L_{Au:Au,Zn:Zn}^{\alpha_{1}-Au_{3}Zn}=-31,715.85+16.239T$	[40]
α_2_-Au_3_Zn	GAu:Znα2-Au3Zn=−19,009+3.074T+0.75G 0Aufcc+0.25GZnhcp 0	[40]
α_3_-Au_3_Zn	GAu:Au:Znα3-Au3Zn=−260,713.32+72.188T+25G 0Aufcc+3GZnhcp 0	[40]
	GAu:Zn:Znα3-Au3Zn=−624,105.44+152.293T+18G 0Aufcc+10GZnhcp 0	[40]
	$L_{Au:Au,Zn:Zn}^{\alpha_{3}-Au_{3}Zn}=-202,141.24+16.494T$	[40]
ε_1_-Au_3_Zn_17_	LAu,Znε1-Au3Zn17=−82,852.97+26.406T 0, LAu,Znε1−Au3Zn17=+58,047.97−20.171T 1	[40]
ε_2_-Au_3_Zn_17_	GAu:Znε2-Au3Zn17=−12,620+0.166T+0.15G 0Aufcc+0.85GZnhcp 0	[40]
β_1_-AuZn	$G_{Au:Zn}^{\beta_{1}-AuZn}=G_{Zn:Au}^{\beta_{1}-AuZn}=-82,852.97+26.40577T$	[40]
	$L_{Au,Zn:Zn}^{\beta_{1}-AuZn}=L_{Zn:Au,Zn}^{\beta_{1}-AuZn}=+58,047.97-20.17125T$	[40]
Au_5_Zn_3_	GAu:ZnAu5Zn3=−192,392.71+32.154T+5G 0Aufcc+3GZnhcp 0	[40]
δ_1_-Au_11_Zn_14_	GAu:Znδ1-Au11Zn14=−618,815.02+25.913T+11G 0Aufcc+14GZnhcp 0	[40]
γ_1_-AuZn_3_	GAu:Au:Au:Znγ1-AuZn3=−255,281.18+13.147T+7G 0Aufcc+6GZnhcp 0	[40]
	GZn:Au:Au:Znγ1-AuZn3=−149,825.99+2.318T+5G 0Aufcc+8GZnhcp 0	[40]
	GAu:Au:Zn:Znγ1-AuZn3=−273,238.57+6.566T+4G 0Aufcc+9GZnhcp 0	[40]
	GZn:Au:Zn:Znγ1-AuZn3=−154,065+20.830T+2G 0Aufcc+11GZnhcp 0	[40]
	$L_{Au,Zn:Au:Au:Zn}^{\gamma_{1}-AuZn_{3}}=L_{Au,Zn:Au:Zn:Zn}^{\gamma_{1}-AuZn_{3}}=-58,164.63$, $L_{Au:Au:Au,Zn:Zn}^{\gamma_{1}-AuZn_{3}}=L_{Zn:Au:Au,Zn:Zn}^{\gamma_{1}-AuZn_{3}}=-107,389.21$	[40]
γ_2_-AuZn_3_	GAu:Znγ2-AuZn3=−78,726.23+5.314T+G 0AuFcc+3GZnHcp 0	[40]
γ_3_-AuZn_3_	GAu:Au:Znγ3-AuZn3=−20,516.36+1.969T+0.28G 0Aufcc+0.72GZnhcp 0	[40]
	GAu:Zn:Znγ3-AuZn3=−9775.48+0.028T+0.12G 0Aufcc+0.88GZnhcp 0	[40]
	$L_{Au:Au,Zn:Zn}^{\gamma_{3}-AuZn_{3}}=-3445.87$	[40]

**Table 6:** Invariant reactions in the Au-Ge-In system.

Invariant Reactions	Type	T (K)	Composition		Reference
			$x_{Au}^{L}$	$x_{Ge}^{L}$	
L + α(Au, In) ↔ fcc(Au) + hcp(Au, In)	U_1_	863	0.765	0.045	This work
L ↔ diamond(Ge) + AuIn + AuIn_2_	E_1_	747	0.444	0.048	This work
		744	0.433	0.035	[28]
L + AuIn ↔ diamond(Ge) +ψ-Au_3_In_2_	U_2_	964	0.586	0.046	This work
L ↔ diamond(Ge) + γ-Au_9_In_4_ +ψ-Au_3_In_2_	E_2_	671	0.641	0.056	This work
L ↔ diamond(Ge) + γ-Au_9_In_4_ + Au_3_In	E_3_	667	0.673	0.080	This work
L ↔ diamond(Ge) + hcp(Au, In) + Au_3_In	E_4_	665	0.691	0.115	This work
L + hcp(Au, In) ↔ diamond(Ge) + fcc(Au)	U_3_	654	0.698	0.259	This work
L ↔ diamond(Ge) + AuIn_2_ + tetragonal(In)	E_5_	429	0.001	0.001	This work
		429	—	—	[28]
L ↔ diamond(Ge) + hcp(Au, In)	m_1_	667	0.696	0.158	This work
L ↔ diamond(Ge) + Au_3_In	m_2_	668	0.679	0.091	This work
L ↔ diamond(Ge) + γ-Au_9_In_4_	m_3_	673	0.649	0.063	This work
L ↔ diamond(Ge) + AuIn	m_4_	751	0.474	0.053	This work
L ↔ diamond(Ge) + AuIn_2_	m_5_	790	0.310	0.067	This work

**Table 7:** Invariant reactions in the Au-Ge-Sb system.

Invariant Reactions	Type	T (K)	Composition		Reference
			$x_{Au}^{L}$	$x_{Ge}^{L}$	
L + rhombohedral (Sb) ↔ diamond (Ge) + AuSb_2_	U_1_	703	0.350	0.140	[33]
		702	0.383	0.137	[15]
		703	0.379	0.137	This work
L ↔ diamond (Ge) + fcc (Au) + AuSb_2_	E_1_	561	0.680	0.150	[33]
		560	0.639	0.166	[15]
		561	0.641	0.166	This work

**Table 8:** Invariant reactions in the Au-Ge-Zn system.

Invariant Reactions	Type	T (K)	Composition		Reference
			$x_{Au}^{L}$	$x_{Ge}^{L}$	
L ↔ β_2_-AuZn + diamond (Ge) + γ_1_-AuZn_3_	E_1_	920	0.368	0.041	This work
L + γ_1_-AuZn_3_ ↔ diamond (Ge) + γ_3_-AuZn	U_1_	841	0.166	0.028	This work
L +β_2_-AuZn ↔ diamond (Ge) + fcc (Au)	U_2_	802	0.610	0.211	This work
L + γ_3_-AuZn ↔ diamond (Ge) + ε_1_-Au_3_Zn_17_	U_3_	747	0.063	0.037	This work
L + ε_1_-Au_3_Zn_17_ ↔ diamond (Ge) + hcp (Zn)	U_4_	693	0.020	0.045	This work
L ↔ diamond (Ge) + β_2_-AuZn	m_1_	944	0.440	0.094	This work
L ↔ diamond (Ge) + γ_1_-AuZn_3_	m_2_	928	0.319	0.041	This work

## Data Availability

All the data that support the findings of this study are included within the article.
